# Detection of tick-borne ‘*Candidatus* Neoehrlichia mikurensis’ and *Anaplasma phagocytophilum* in Spain in 2013

**DOI:** 10.1186/1756-3305-7-57

**Published:** 2014-01-31

**Authors:** Ana M Palomar, Lara García-Álvarez, Sonia Santibáñez, Aránzazu Portillo, José A Oteo

**Affiliations:** 1 Departamento de Enfermedades Infecciosas, Hospital San Pedro-CIBIR, Center of Rickettsioses and Arthropod-Borne Diseases, C/ Piqueras, N° 98, 26006 Logroño, La Rioja, Spain

**Keywords:** **‘***Candidatus* Neoehrlichia mikurensis’, *Anaplasma phagocytophilum*, *Ixodes ricinus*, Spain

## Abstract

**Background:**

‘*Candidatus* Neoehrlichia mikurensis’ is a tick-borne bacteria implicated in human health. To date, ‘*Ca.* Neoehrlichia mikurensis’ has been described in different countries from Africa, Asia and Europe, but never in Spain. However, according to the epidemiological features of the main vector in Europe, *Ixodes ricinus*, its circulation in our country was suspected.

**Methods:**

A total of 200 *I. ricinus* ticks collected in the North of Spain were analyzed. DNAs were extracted and used as templates for PCRs targeting fragment genes for *Anaplasma*/*Ehrlichia* detection. The amplified products were sequenced and analyzed.

**Results:**

‘*Ca.* Neoehrlichia mikurensis’ was amplified in two specimens. Furthermore, *Anaplasma phagocytophilum* was detected in 61 samples analyzed.

**Conclusions:**

The detection of ‘*Ca.* Neoehrlichia mikurensis’ in *I. ricinus* ticks from Spain indicates its circulation and the potential risk of contracting a human infection in this country.

## Background

*‘Candidatus* Neoehrlichia mikurensis’ is an obligate intracellular bacterium member of the *Anaplasmataceae* family. It was first isolated from wild rats (*Rattus norvegicus*) and *Ixodes ovatus* ticks in the Mikura Island, Japan [1]. It was classified as a new genus (*Neoehrlichia*) added to those already known of the *Anaplasmataceae* family: *Ehrlichia*, *Anaplasma*, *Neorickettsia*, *Aegyptianella* and *Wolbachia*[1].

The presence of ‘*Ca.* Neoehrlichia mikurensis’ in rodents and ticks has been notified from different countries of Europe, Asia and Africa in the last decade [2,3]. In Europe, it has been mostly detected in *Ixodes ricinus*, although it has been associated to other tick species in other continents. *I. ricinus*, endemic in the North of Spain, is responsible for most human tick bites. It acts as vector of different human pathogens, such as *Borrelia burgdorferi* sensu lato (s.l.), *Anaplasma phagocytophilum* -formerly *Ehrlichia phagocytophila*- or different *Rickettsia* spp., protozoa and arboviruses. However, the risk of infections with ‘*Ca.* Neoehrlichia mikurensis’ to human health remains unclear in southern Europe.

The first implication of the bacterium in human pathology was reported in Sweden in 2010 [4]. Subsequently, seven new human cases severely affected by ‘*Ca.* Neoehrlichia mikurensis’ infections have been notified from Europe [5-8]. Several human cases have also been described in China [2].

*‘Ca.* Neoehrlichia mikurensis’ has not been previously described in Spain. However, according to the epidemiological features of the main vector, *I. ricinus*, in which the bacterium has been mostly detected in Europe, its circulation in our country was suspected.

## Methods

In the routine analysis of tick-borne pathogens performed in the Center of Rickettsioses and Arthropod-borne Diseases (Logroño, Spain), 200 *I. ricinus* ticks collected from cows were tested. Samples were obtained in two different locations of La Rioja (Spain): Tobía (42°18’N; 2°48’W) and Jubera (42°18’N; 2°17’W) in April 2013. A total of 50 males and 50 engorged females from each location were processed. Ticks were kept at -80°C until DNA extraction with Qiagen DNeasy Blood & Tissue Kit, according to the manufacturer’s instructions (Qiagen, Hilden, Germany).

All DNA extracts were used as templates for two nested PCRs targeting fragment genes for *Anaplasma*/*Ehrlichia* detection. Furthermore, a simple PCR was performed to confirm the amplification of species never detected in the area (Table 1) [9-11]. Two negative controls, one of them containing water instead of template DNA and the other with template DNA but without primers, as well as a positive control of *A. phagocytophilum* were included in all PCR assays. Amplification products were sequenced, and nucleotide sequences were compared with those available in GenBank by using a Basic Local Alignment Search Tool (BLAST) search (http://www.ncbi.nlm.nih.gov/blast).

**Table 1:** PCR primer pairs used in this study

**Gene target**	**Primer name**	**Primer sequence 5’→ 3’**	**Amplified fragment (bp)**	**Annealing temperature (ºC)**	**Reference**
*groESL* heat shock operon of *Anaplasma* spp. (nested)	HS1a	AITGGGCTGGTAITGAAAT	1350	48	[9]
	HS6a	CCICCIGGIACIAIACCTTC			
	HS43	AT(A/T)GC(A/T)AA(G/A)GAAGCATAGTC	1297	55	
	HSVR	CTCAACAGCAGCTCTAGTAGC			
16S rRNA (nested)	ge3a	CACATGCAAGTCGAACGGATTATTC	932	55	[10]
	ge10r	TTCCGTTAAGAAGGAT CTAATCTCC			
	ge9f	AACGGATTATTCTTTATAGCTTGCT	546	55	
	ge2	GGCAGTATTAAAAGCAGCTCCAGG			
16S rRNA EHR	EHR 16SD	GGTACCYACAGAAGAAGTCC	345	55	[11]
	EHR 16SR	TAGCACTCATCGTTTACAGC			

## Results and discussion

Two nucleotide sequences of *groESL* fragment gene (1%) corresponding to 2 male tick specimens collected in Tobía showed 100% identity with ‘*Ca.* Neoehrlichia mikurensis’. They were identical to the one detected in two patients in Germany [5]. None of them yielded positive results when PCR tests for 16S rRNA were performed. For this reason, a different fragment of the 16S rRNA gene (EHR) was investigated to confirm our previous results. Nucleotide sequences of both samples were identical to each other and showed 100% identity with more than one sequence of ‘*Ca.* Neoehrlichia mikurensis’ (Table 2). In our laboratory we had never worked with ‘*Ca.* Neoehrlichia mikurensis’ before, so no contamination with this bacterium was possible.

**Table 2:** ***Anaplasmataceae *** **species detected in *Ixodes ricinus *** **removed from cows (N = 200) in La Rioja (North of Spain)**

**Bacterium (no.)**	***groESL***				**16S rRNA**				**16S rRNA-EHR**			
	**Ticks, number and stage**		**Genbank accession no.**	**Identity (%)**	**Ticks, number and stage**		**Genbank accession no.**	**Identity (%)**	**Ticks, number and stage**		**Genbank accession no.**	**Identity (%)**
	**Tobía**	**Jubera**			**Tobía**	**Jubera**			**Tobía**	**Jubera**		
*‘Ca.* N. mikurensis’ (2)	2 M		EU810407	100					2 M		JQ675350	100
*A. phagocytophilum* (61)												
Human pathogenic variant (8)	1 M, 2 F	2 M, 2 F	U72628	99.9-100	1 F*	2 F	U02521	100				
Non-pathogenic variants (53)	1 M		AF478558	100	1 M		JN181071	100				
	1 M		AF478563	100								
	1 F		AY281831	100		1 F	EU839849	100				
	2 F	2 M, 7 F	EU246959	99.8-100	1 F	1 M, 4 F	JN181071	100				
	5 F	2 M, 29 F	AF548385	99.8-100	2 F	1 M, 11 F^†^	JN181071	99.9-100				
		1 M	AY281830	100		1 M	JN181071	100				

No.: number*; ‘Ca.* N. mikurensis’: ‘*Candidatus* Neoehrlichia mikurensis’; *A. phagocytophilum*: *Anaplasma phagocytophilum*; M: Male; F: Female; *: only amplified with 16S rRNA fragment gene; ^†^: Two of them only amplified with 16S rRNA fragment gene.

On the other hand, *A. phagocytophilum* was detected in 61 samples (30.5%) of this study. Specifically, 8 specimens (4%) showed maximum identity with the human pathogenic variant, and 53 (26.5%) with non-pathogenic variants (Table 2).

In this study, ‘*Ca.* Neoehrlichia mikurensis’ DNA was detected in two ticks from La Rioja (Spain) during 2013 but we do not know if this bacterium has been previously circulating in our area. Anyway, this infection may be underdiagnosed in our media. In addition, according to the recent finding of several human cases due to this bacterium, mainly in immunocompromised patients, physicians should be aware of the risk for those patients in the affected area. Moreover, infections and fever of unknown origin are common in immunocompromised patients and the responsible pathogen is not isolated in most cases [7]. The detection of ‘*Ca.* Neoehrlichia mikurensis’ and the features of the European human cases suggest that this microorganism is likely causing disease in our country too.

The prevalence of *A. phagocytophilum* in the studied area has been previously reported [12]. According to our results, the high prevalence of the bacterium in the engorged females collected in Jubera should be noted (40 out of 50 specimens, 80%). This could be due to the fact that all the female specimens processed were engorged on cows, hosts that are potential amplifiers of the bacterium [13].

## Conclusions

*‘Ca.* Neoehrlichia mikurensis’ has been detected in *I. ricinus* ticks removed from cows in Spain. *A. phagocytophilum* was amplified in 61 out of 200 samples (8 of them corresponding to the human pathogenic variant). Our results suggest that human infections by ‘*Ca.* Neoehrlichia mikurensis’ might be undiagnosed in this country. Further research should be carried out to study the epidemiology of the bacterium as well as to be aware of possible human cases.

## Competing interests

The authors declare they have no competing interests.

## Authors’ contributions

Designed the study: **AMP, AP, JAO.** Collected and identified ticks: **AMP**. Processed samples: **AMP, LGA**. Performed PCR: **AMP, LGA, SS**. Analyzed sequences: **AMP, SS, AP**. Analyzed the data: **AMP, AP, JAO**. Wrote the paper: **AMP, LGA, SS, AP, JAO.** All authors read and approved the final version of the manuscript.
